# Ethnic/Race Diversity and Diabetic Kidney Disease

**DOI:** 10.3390/jcm4081561

**Published:** 2015-07-31

**Authors:** Vasantha Muthu Muthuppalaniappan, Muhammad Magdi Yaqoob

**Affiliations:** 1 Queen Mary University of London, London EC1M 6BQ, UK; E-Mail: v.m.muthuppalaniappan@qmul.ac.uk; 2 Barts Health NHS Trust and Queen Mary University of London, London EC1M 6BQ, UK

**Keywords:** diabetic kidney disease, ethnicity, race

## Abstract

Ethnicity and race are often used interchangeably in the literature. However, the traditional definition of race and ethnicity is related to biological (bone structure and skin, hair, or eye color) and sociological factors (nationality, regional culture, ancestry, and language) respectively. Diabetes mellitus (DM) is a huge global public health problem. As the number of individuals with Type 2 DM grows, the prevalence of diabetic kidney disease (DKD), which is one of the most serious complications, is expected to rise sharply. Many ethnic and racial groups have a greater risk of developing DM and its associated macro and micro-vascular complications.

## 1. Introduction

Even though ethnicity and race are often used interchangeably in medical literature there are clear differences in the traditional definitions of these two words. Ethnicity refers to a group of human beings with real or presumed common genealogy or ancestry and shared cultural and or linguistics and or religious traits. On the contrary race refers to groups of population with different physical characteristics, which usually result from genetic ancestry. Races are usually distinguished by skin colour and facial type. Racial categories result from a shared genealogy due to geographical isolation. In the modern world this isolation has been broken down and racial groups have mixed. In this review we have used these two terms as they appear in literature in the context of diabetes mellitus (DM) and diabetic kidney disease (DKD).

DKD is one of the leading causes of end stage renal disease in the world (ESRD). Up to 20%–40% of patients who suffer from DM will develop DKD [1]. The risk factors currently identified for the development and progression of DKD include duration of DM, poor glycaemic control and hypertension [2]. However there is growing body of evidence to suggest that individuals from certain ethnic and racial backgrounds have higher susceptibility to develop both DM and its associated macro and micro-vascular complications.

## 2. Epidemiological Evidence for Ethnicity/Racial Predisposition towards DKD

The prevalence of DM is much higher among Black and South Asian populations in comparison to Whites. Mortality is also 3.5 times higher in South Asian and Black populations with DKD compared to Whites in England and Wales [3]. Most albeit small population studies have demonstrated that South Asians and Blacks with DM are more likely to develop DKD and have a faster rate of progression towards ESRD than their White counterparts [4,5]. A *post hoc* analysis of the Reduction of Endpoints in NIDDM with the Angiotensin II Antagonist Losartan (RENAAL) study showed that the Hispanic and Asian populations had a higher risk for ESRD and higher baseline albuminuria level than the Black and White population [6]. The UK Prospective Diabetes Study (UKPDS) showed that Indian-Asian patients were more likely to develop nephropathy than the Whites, with Indian-Asian ethnicity being an independent predictor of albuminuria and renal impairment [7].

Recently a large, multi-ethnic East London, United Kingdom (UK) cohort of DM patients (*n* = 34,359) and high-recorded levels of self-reported ethnicity reported the effect of ethnicity on the prevalence and severity of diabetes mellitus and associated chronic kidney disease (CKD) [8]. The prevalence of DM was 3.5% for Whites, 11% for South Asians and 8% for Black groups [9]. The prevalence of CKD (stages 3–5) among diabetics was 18%. CKD stage 3 was more prevalent in Whites compared to South Asians and Blacks [9]. The severity of CKD stages 4 and 5 was associated with Black and South Asian ethnicity compared to Whites [9]. Proteinuria was also more prevalent in Black and South Asian patients compared to White patients.

Significant disparities existed between the major ethnic and racial groups in both disease prevalence and management in terms of achieved targets recommended in guidelines, which may in part explain these differences. The disparities currently seen across ethnic and racial groups are clearly influenced by genetic susceptibility, socioeconomic status, lifestyle choices and/or environmental exposure. Ethnic minority groups may be more prone to metabolic syndrome that may predispose them to microalbuminuria or macroalbuminuria once diabetes develops [10]. Genetic differences, such as angiotensin converting enzyme (ACE) gene polymorphisms could account for the relative lack of response to ACE-inhibitors (ACEi) in certain ethnic groups. Currently there are no national CKD guidelines that include ethnicity as a risk factor for CKD and as a result of this the management of DKD across ethnic minorities are similar to that of the White population. Adopting a uniform approach in the UK across all patients with DKD may paradoxically disadvantage subgroups that might benefit from a more personalized approach.

## 3. Effect of Ethnicity and Race in the Community

The same group of investigators in a separate retrospective study over a period of 5 years investigated the effect of ethnicity and race on the progression of kidney disease among patients with DKD managed in a community setting. This community-based cohort study included 3855 people with DM of White, Black or South Asian race/ethnicity and an estimated glomerular filtration rate (eGFR) of <60 mL/min. The mean annual adjusted decline in eGFR for the entire cohort was 0.85 mL/min [11]. The rate decline was statistically greater in the South Asian group (−1.01 mL/min) compared with the White group (−0.70 mL/min) (*p* = 0.001) [11]. As expected, for those individuals with proteinuria at baseline, the annual decline was greater at 2.05 mL/min, with both South Asian and Black groups having a significantly faster rate of decline than the White group [11]. Interestingly, in the community setting alleged renoprotective effects of ACEi and/or angiotensin receptor blockers (ARBs) were not borne out. Reassuringly the rate of decline in eGFR for patients with DM and early stages of CKD managed in primary care setting, was less than previously thought and approximated to an age-related annual decline of 1 mL/min [11]. More importantly this study identified that patient with proteinuria and particularly those of South Asian and Black ethnicity/race are high-risk and might benefit from additional monitoring in a specialist clinic.

## 4. Ethnicity/Racial Influence on Specialist Management of DKD

In order to investigate the effect of specialist management on the rate of progression of CKD in patients with DM, stratified by ethnicity and race, a prospective cohort study of patients managed in a tertiary hospital setting was undertaken. All new patients referred with DKD between 2000 and 2007 were included with a mean follow up duration greater than 5 years. 329 patients (208 men) were studied comprising 149 South Asian, 105 White and 75 Black patients [12]. The overall annual decline in eGFR was −1.69 mL/min in the entire cohort [12]. In the unadjusted linear regression analysis the annual decline in eGFR (mL/min) in White, Black and South Asian groups was −1.78, −2.02 and −1.51 respectively [12]. However, following adjustment for age at baseline, gender, presence or absence of vascular disease, drug treatment, presence or absence of proteinuria at baseline and baseline eGFR, no significant difference was observed in the rate of decline in eGFR between ethnic groups. In addition, there was no difference between ethnic groups in either all-cause mortality or the number of individuals developing end stage kidney failure. ACE-I or ARB use, and glycated haemoglobin (HbA1C) were similar at baseline and throughout the study period. Male gender, severity of renal impairment and blood pressure control were the only independent predictors of progression. It was concluded that ethnicity is not an independent risk factor for the progression of renal failure in patients with DM and CKD if managed in a specialist clinic setting subject to achieving similar glycaemic and blood pressure control [12].

## 5. Conclusions

The underlying causes of clear differences in the incidence of DM and its associated complications in different ethnic and racial groups are possibly due to different cultural traits, life style choices and more importantly attitudes to health education/adherence to dietary advice and medications. The genetic susceptibility to DKD may vary in racial/ethnic groups as suggested by a recent meta-analysis of 42 studies evaluating genetic variants involved in the renin angiotensin system which showed that the angiotensin enzyme gene polymorphism was associated with DKD in the Asian subgroup but not in Europeans [13]. However, it is generally believed that the direct genetic component in the development of DKD is small. It is highly likely that diabetic milieu in the context of different life style, cultural and racial traits leads to modifiable epigenetic changes and associated functional genomic abnormalities. We envisage that ongoing studies will hopefully identify molecular epigenetic signature, which could be used both in risk stratification and personalized therapy rather than one size fits all approach practiced currently.
